# Comparative Analysis of Diet Composition and Dietary Diversity in Wild Przewalski’s Gazelle and Sympatric Domestic Sheep Around Qinghai Lake, Western China

**DOI:** 10.3390/ani16213426

**Published:** 2026-10-29

**Authors:** Lili Hou, Shuaipeng Xu, Ming Xu

**Affiliations:** 1 Faculty of Geographic Science and Engineering, College of Geographical Sciences, Henan University, Zhengzhou 450046, China; houlili@henu.edu.cn; 2 Guangdong-Hong Kong Joint Laboratory for Carbon Neutrality, Jiangmen Laboratory of Carbon Science and Technology, Jiangmen 529199, China; xushuaipeng@hkustgz-jcl.ac.cn

**Keywords:** Przewalski’s gazelle, domestic sheep, diet composition, dietary overlap, niche breadth, Qinghai Lake Basin

## Abstract

Understanding dietary relationships between wild ungulates and sympatric livestock is important for conserving threatened species in alpine grassland ecosystems. Przewalski’s gazelle (*Procapra przewalskii*) is an endangered ungulate endemic to the Qinghai Lake Basin, but its dietary relationship with sympatric domestic sheep across multiple subpopulations remains poorly understood, particularly at finer taxonomic scales. Using fecal microhistology of April 2023 samples from eight study sites during the late winter grazing season, we compared dietary composition, diversity, evenness, niche breadth, and overlap at the family, genus, and species levels. More plant taxa were identified in the fecal samples of Przewalski’s gazelle than in those of domestic sheep, and estimates of dietary diversity, evenness, and niche breadth were generally higher for gazelles. Both herbivores relied largely on graminoids, indicating a shared dietary foundation. However, dietary overlap was higher at the family level (Pianka’s index = 0.830–0.976) than at the genus (0.718–0.946) and species (0.629–0.946) levels, with substantial variation among subpopulations. These results suggest a shared broad dietary foundation but apparent differentiation in resource use at finer taxonomic scales during late winter. These findings can inform Przewalski’s gazelle conservation and grassland management in the Qinghai Lake Basin.

## Simple Summary

Przewalski’s gazelle is an endangered ungulate found only in the Qinghai Lake Basin, where it often shares grazing areas with domestic sheep. To better understand how these two herbivores use food resources when they occur in the same areas, we compared their diets across eight subpopulations using fecal samples collected during the late winter grazing season. Both herbivores relied mainly on similar major plant groups, but Przewalski’s gazelle fecal samples contained a wider range of identified plant taxa and showed higher estimated dietary diversity than domestic sheep samples. The two herbivores appeared more similar when plants were grouped into broad categories, but differences between their diets became clearer when plants were identified more precisely. The extent to which their diets overlapped also varied among subpopulations. These findings improve our understanding of how Przewalski’s gazelle and domestic sheep share food resources and may help inform conservation and grassland management in the Qinghai Lake Basin.

## 1. Introduction

Competition for resources between wild ungulates and domestic livestock is an important ecological issue in alpine grassland ecosystems [1,2]. As human activities and pastoral expansion intensify, spatial and dietary overlap between wild herbivores and livestock may increase, potentially heightening resource pressure on endangered species [3,4]. Understanding how wild ungulates and livestock use food resources under such shared-use conditions is therefore important for assessing their dietary relationships in grazing landscapes with substantial resource overlap and potential competition [5].

Przewalski’s gazelle (*Procapra przewalskii*) is an ungulate species listed as Endangered (EN) on the IUCN Red List. Historically distributed across Qinghai, Gansu, Ningxia, and Inner Mongolia, the species is now largely restricted to the Qinghai Lake Basin, where the remaining populations persist in small, fragmented, and isolated subpopulations [6,7,8]. Its population was estimated at fewer than 300 individuals in the 1990s [8], whereas a population survey recorded 4078 individuals in 2020, although some subpopulations continued to decline [6]. These subpopulations continue to be threatened by habitat fragmentation [9] and other anthropogenic pressures [10,11,12]. Previous studies have shown that fencing and livestock-related disturbances can restrict the movements of Przewalski’s gazelle [11,12] and influence its daily foraging activity, with feeding often concentrated in the early morning and late afternoon [7]. Across most of its range, the species occurs sympatrically with domestic sheep, and livestock grazing has long been considered an important factor potentially influencing habitat use and population persistence [6,13].

In the Qinghai Lake Basin, domestic sheep are generally herded on natural grasslands, and fencing does not necessarily result in complete spatial separation from free-ranging Przewalski’s gazelles [11,12]. Adult Przewalski’s gazelles weigh approximately 17–32 kg [14], whereas yearling male Tibetan sheep in the Qinghai Lake Basin weigh approximately 30 ± 2 kg [15]. Their broadly comparable body masses and shared use of winter grazing pastures highlight the potential for competition over forage resources [16], particularly during the late winter grazing season in April, when forage availability may be limited. However, how the two herbivores differ in their use of food resources under sympatric conditions remains poorly understood.

Despite longstanding conservation concern, previous studies on Przewalski’s gazelle have focused mainly on population dynamics [17,18] and habitat selection [9,19], whereas its dietary ecology has received comparatively less attention. Existing dietary studies have addressed topics such as seasonal diet variation [20] and dietary overlap with domestic livestock in local subpopulations [21]. A study in the Upper Buha River Valley also documented sympatry and substantial dietary overlap between Przewalski’s gazelle and Tibetan gazelle (*Procapra picticaudata*), suggesting that differences in their foraging areas may help reduce competition [16]. However, previous dietary studies have generally been limited to one or a few local subpopulations and have not systematically examined dietary relationships across multiple subpopulations and taxonomic levels [16,21]. Consequently, it remains unclear whether a general regional pattern in the dietary relationship between Przewalski’s gazelle and domestic sheep emerges across multiple sympatric subpopulations, and whether differences in resource use become more apparent at finer taxonomic scales beyond the broad overlap observed in major food groups.

To address these gaps, we investigated eight sympatric subpopulations of Przewalski’s gazelle and domestic sheep around Qinghai Lake and systematically compared their diets at the family, genus, and species levels. These eight subpopulations collectively accounted for approximately 87.2% (3557 of 4078 individuals) of the population estimated in 2020 [6]. Specifically, we aimed to (1) compare dietary taxonomic coverage between the two herbivores across taxonomic levels; (2) evaluate differences in dietary diversity, evenness, and niche breadth; and (3) quantify dietary overlap at the family, genus, and species levels. By integrating comparisons across multiple sympatric subpopulations and taxonomic levels, this study aims to clarify the dietary relationship between Przewalski’s gazelle and domestic sheep in shared grazing landscapes and to provide an ecological basis for gazelle conservation and the management of sympatric grasslands in the Qinghai Lake Basin.

## 2. Materials and Methods

### 2.1. Study Area

**Figure 1:**
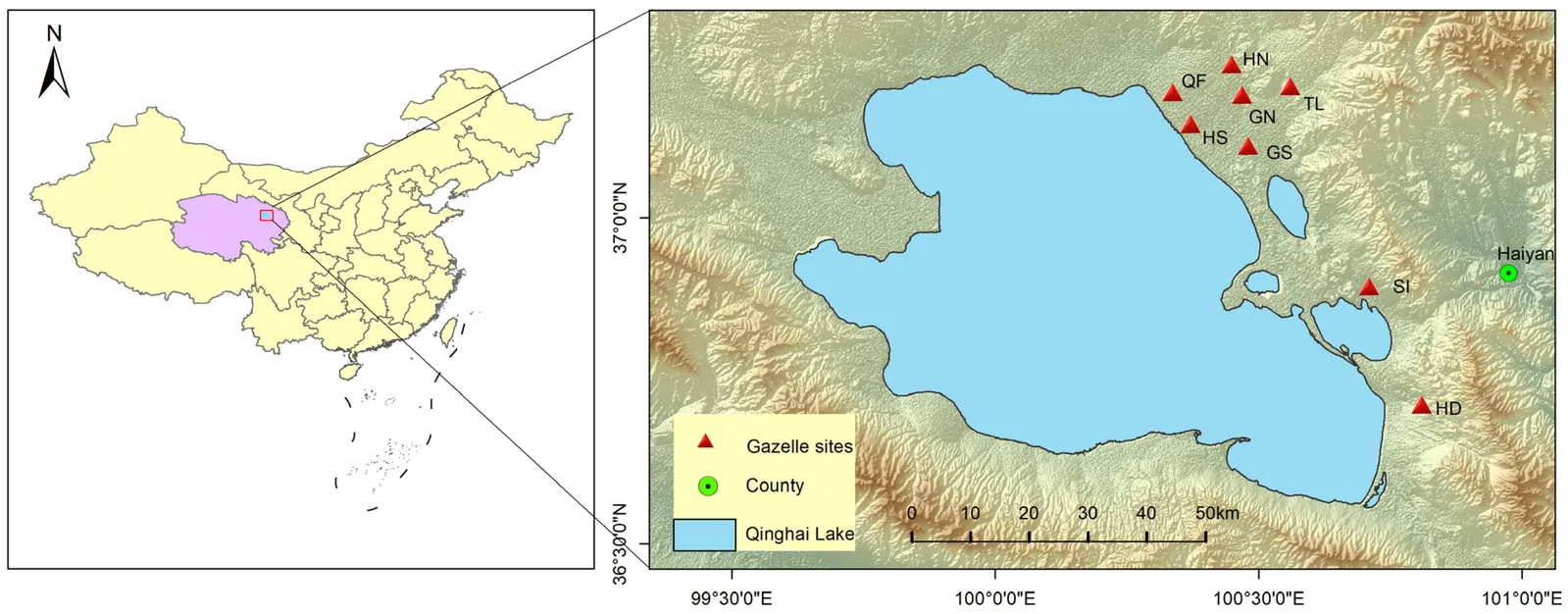
Study sites of the eight geographically distinct Przewalski’s gazelle subpopulations in the Qinghai Lake Basin. In the locator map (**left**), Qinghai Province is highlighted in purple, while other provincial-level administrative regions are shown in pale yellow. The red box indicates the study area, and the connecting lines link the locator map to the enlarged map (**right**). The dashed line in the South China Sea area indicates the boundary line shown on the base map. In the enlarged map (**right**), red triangles indicate gazelle sampling sites, the green circle indicates Haiyan County, and the blue area represents Qinghai Lake. The surveyed locations included Qinghai Lake Farm No. 1 Branch Factory (QF), the northern (HN) and southern (HS) sides of the Hargai Railway, Talexuanguo (TL), the northern (GN) and southern (GS) sides of the Ganzihe Railway, Sand Island (SI), and Hudong (HD). QF represents the Qinghai Lake Farm No. 1 Branch Factory area, which is a geographic name rather than a captive facility.

The study was conducted in the Qinghai Lake Basin on the northeastern Qinghai–Tibet Plateau (97°05′–101°02′ E, 35°05′–37°05′ N). The region has a typical continental plateau climate, with a mean annual temperature of −0.8 to 1.1 °C and annual precipitation of 327–423 mm [22]. Alpine meadows and steppes are the predominant vegetation types in the region, with *Kobresia humilis*, *Achnatherum splendens*, and *Stipa purpurea* among the common plant species [23,24]. We selected eight geographically distinct free-ranging wild Przewalski’s gazelle subpopulations around Qinghai Lake: Qinghai Lake Farm No. 1 Branch Factory (QF), Talexuanguo (TL), the northern (GN) and southern (GS) sides of the Ganzihe Railway, the northern (HN) and southern (HS) sides of the Hargai Railway, Hudong (HD), and Sand Island (SI). Here, Qinghai Lake Farm No. 1 Branch Factory (QF) refers to the geographical name of the sampling site rather than a commercial farm, livestock production facility, or captive breeding area. Domestic sheep co-occurred with Przewalski’s gazelle at all selected sites, allowing paired comparisons between the two herbivores within each subpopulation [7,13]. By focusing on sites where the two herbivores occurred sympatrically, this study was designed to examine their dietary relationships under shared grazing conditions (Figure 1).

### 2.2. Fecal Sample Collection

Field surveys were conducted in April 2023 at all eight study sites. This period corresponded to the latter part of the winter grazing season, when Przewalski’s gazelle and domestic sheep commonly shared winter grazing areas [11], thereby providing a suitable window for assessing their dietary relationships under sympatric conditions. Fecal samples from Przewalski’s gazelle and domestic sheep were collected at each site [25]. At each site, 5–8 spatially separated fecal samples were collected for each herbivore species across multiple locations to improve spatial representativeness and reduce the likelihood of repeatedly sampling the same individuals. In total, 59 fecal deposits from Przewalski’s gazelle and 48 from domestic sheep were sampled across the eight study sites (Table S1). Przewalski’s gazelle feces were typically spindle-shaped and deposited in piles, whereas domestic sheep feces were more rounded and commonly scattered along grazing paths. The two fecal types were distinguished in the field based on pellet morphology, deposition pattern, and associated field signs [16]. Representative photographs of both fecal types are provided in Figure S1.

### 2.3. Fecal Microhistological Analysis and Diet Composition

Diet composition was determined using fecal microhistology following a modified sodium hypochlorite digestion and clearing method [26]. At each study site, fecal deposits collected from each herbivore species were combined to form a single composite sample. Specifically, ten fecal pellets were selected from each spatially separated fecal deposit and pooled within each herbivore species and site, resulting in one composite dietary sample per herbivore per site. The composite fecal material was dried at 60 °C for 48 h after arrival at the laboratory, thoroughly ground and homogenized, and sieved. Particles between 40 and 100 mesh were retained for analysis. A 0.5 g subsample was taken from each homogenized composite sample and cleared in 10% sodium hypochlorite (Shanghai Lingfeng Chemical Reagent Co., Ltd., Shanghai, China) for 4–8 h, stained with safranin (Tianjin Kermel Chemical Reagent Co., Ltd., Tianjin, China) for 30 min, and mounted on microscope slides.

Reference slides used for microhistological identification were prepared from aboveground parts of local vascular plant species collected from the same study area during the growing season (July), when diagnostic morphological features were fully developed. Plant materials were identified to species level by a botanist and processed into reference slides for comparison with plant fragments observed in fecal samples. Representative examples of plant epidermal microstructures and corresponding fragments identified in feces are shown in Table S2. These reference slides were used for taxonomic identification of plant fragments in fecal samples and were not intended to represent seasonal plant availability in the field.

For each pooled sample, a single trained observer examined 300 microscopic fields under a Leica DM2500 microscope (Leica Microsystems, Wetzlar, Germany) at 100× magnification. The number of microscopic fields was selected to provide adequate observation coverage [27], while the use of a single trained observer helped maintain consistency in taxonomic identification and minimize inter-observer variation [28]. Identified plant fragments were assigned to the family, genus, or species level whenever possible.

Fragment frequency and relative density (RD) were calculated using the frequency-conversion method [25] as follows:

(1) $$F=100(1-e^{-D})$$

(2) $$RD_{i}=\frac{D_{i}}{\sum D_{i}}\times 100$$

where *F* is the frequency of occurrence (%) of identifiable epidermal fragments, *D* is the estimated mean density of identifiable epidermal fragments per microscopic field, *D_i_* is the fragment density of taxon *i*, and $\sum D_{i}$ is the summed fragment density of all identified taxa. RD was used to describe the relative representation of identifiable plant fragments in fecal samples, rather than the actual biomass of plant material ingested. To compare dietary taxonomic coverage between the two herbivores, we recorded the numbers of plant families, genera, and species detected within each subpopulation.

### 2.4. Diet Diversity and Niche Breadth

To quantify dietary diversity and resource use breadth, we calculated richness (*S*), Shannon diversity (*H*′), Pielou’s evenness (*J*), and Levins’ niche breadth (*B*) at the family, genus, and species levels [25]:

(3) $$H^{\prime }=-\sum_{i=1}^{S}p_{i}\ln p_{i}$$

(4) $$J=\frac{H^{\prime }}{\ln S}$$

(5) $$B=\frac{1}{\sum_{i=1}^{S}p_{i}^{2}}$$

where *p*_*i*_ is the proportional contribution of taxon *i* in the diet, calculated from its relative density (RD), and *S* is the number of taxa recorded at the corresponding taxonomic level. Higher values of *H*′, *J*, and *B* indicate greater dietary diversity, more even resource use, and broader niche breadth, respectively. For the pooled summaries, the relative density of each taxon was averaged across the eight subpopulations, with each subpopulation weighted equally, and *H*′ and *J* were then calculated from the resulting dietary composition.

### 2.5. Dietary Overlap and Classification of Shared Forage Species

We quantified dietary overlap between Przewalski’s gazelle and domestic sheep using Pianka’s index [29]:

(6) $$O_{\mathrm{jk}}=\frac{\sum_{i=1}^{S}p_{\mathrm{ij}}p_{\mathrm{ik}}}{\sqrt{\left(\sum_{i=1}^{S}p_{\mathrm{ij}}^{2}\right)\left(\sum_{i=1}^{S}p_{\mathrm{ik}}^{2}\right)}}$$

where *p_ij_* and *p_ik_* are the relative contributions of taxon *i* to the diets of herbivores *j* and *k*, respectively, and *S* is the total number of taxa recorded across the two herbivores at the corresponding taxonomic level within a given study site. Pianka’s index ranges from 0 (no overlap) to 1 (complete overlap) [29]. Dietary overlap was calculated separately at the family, genus, and species levels for each study site.

We also recorded the numbers of shared and unique taxa between the two herbivores at the family, genus, and species levels. For descriptive purposes, plant species were operationally classified according to their occurrence across study sites and mean relative density (RD) across the eight sites, with non-detections treated as zero when calculating mean RD. Species recorded in at least five of the eight sites in both herbivores and showing a mean RD of at least 2% in at least one herbivore were defined as shared core resources. Species detected in both herbivores across the eight study sites but not meeting these criteria were classified as shared secondary resources. Species detected only in Przewalski’s gazelle or only in domestic sheep across the eight study sites were classified as gazelle-exclusive or sheep-exclusive, respectively. Differences in mean RD between the two herbivores were reported descriptively to indicate variation in the relative use of individual shared forage species.

### 2.6. Statistical Analysis

Each sympatric subpopulation was treated as one paired sampling unit, and all comparisons between Przewalski’s gazelle and domestic sheep were therefore conducted at the subpopulation level. To compare taxonomic coverage, dietary diversity, evenness, and niche breadth between the two herbivores at the family, genus, and species levels, the distributions of paired differences were assessed for normality using the Shapiro–Wilk test. The paired differences of all tested metrics met the assumption of normality (all *p* > 0.05); therefore, two-tailed paired-samples *t*-tests were used for all comparisons. Statistical significance was set at *p* < 0.05. Primary calculations were conducted in OriginPro 2025 (OriginLab Corporation, Northampton, MA, USA), and supplementary statistical analyses were performed in R version 4.4.1 (R Foundation for Statistical Computing, Vienna, Austria).

## 3. Results

### 3.1. Diet Composition at Family, Genus, and Species Levels

Across the eight sympatric subpopulations, dietary taxonomic richness was generally higher in Przewalski’s gazelle than in domestic sheep at the family, genus, and species levels (Table 1). At the family level, Przewalski’s gazelle used 81.82–100.0% of the total number of families detected in either herbivore within each subpopulation, whereas domestic sheep used 63.64–91.67%. At the genus level, the corresponding proportions were 83.33–100.0% for Przewalski’s gazelle and 52.38–86.67% for domestic sheep. At the species level, Przewalski’s gazelle used 85.00–100.0% of the total number of species detected in either herbivore within each subpopulation, whereas domestic sheep used 54.55–82.35%. Subpopulation-level taxonomic richness was significantly higher in Przewalski’s gazelle than in domestic sheep at the family (*t*_7_ = 4.97, *p* = 0.0016), genus (*t*_7_ = 4.71, *p* = 0.0022), and species (*t*_7_ = 5.95, *p* = 0.0006) levels. When taxa detected across all eight subpopulations were considered as a regional union, 18 families, 36 genera, and 41 species were recorded in the diet of Przewalski’s gazelle, compared with 17 families, 32 genera, and 34 species in domestic sheep. The corresponding totals for domestic sheep were 94.44%, 88.89%, and 82.93% of those recorded for Przewalski’s gazelle at the family, genus, and species levels, respectively.

**Figure 2:**
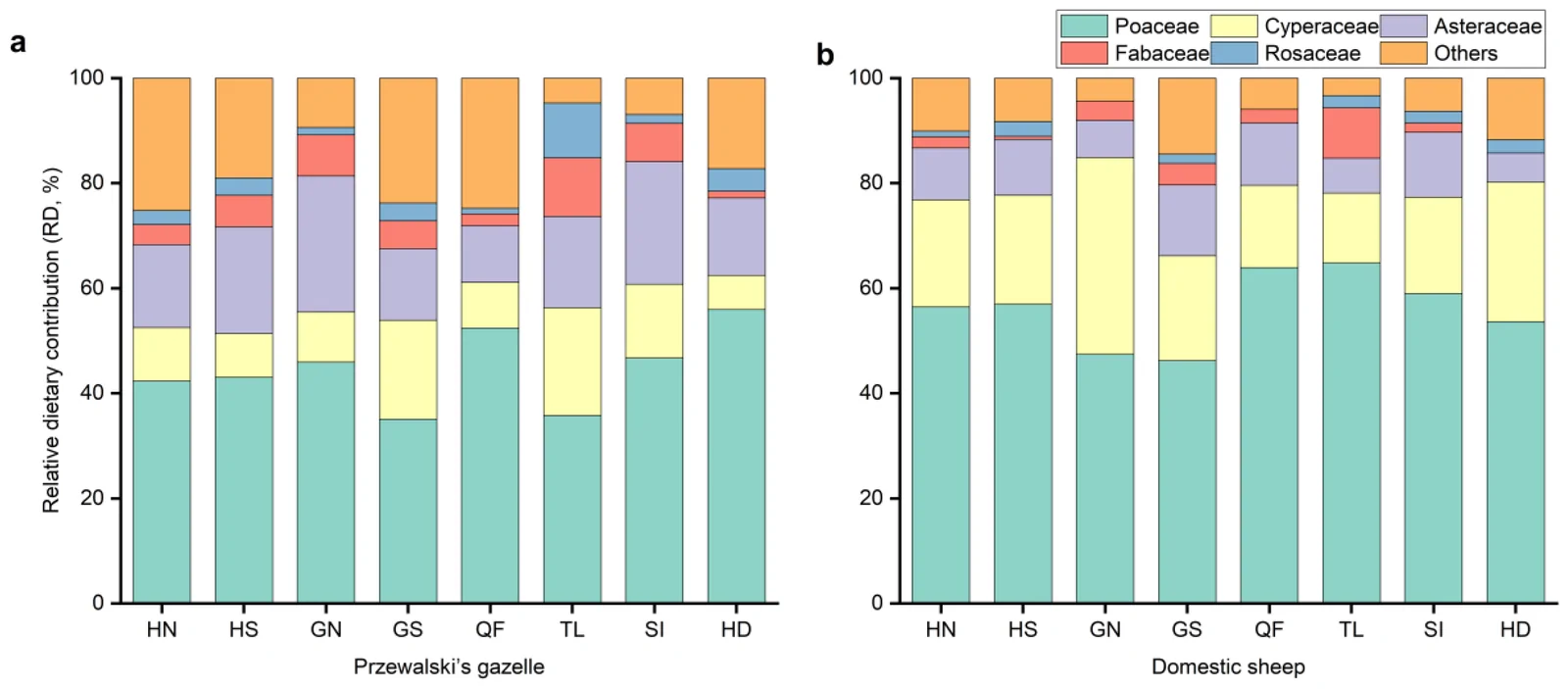
Family-level dietary composition of Przewalski’s gazelle (**a**) and domestic sheep (**b**) across eight sympatric subpopulations around Qinghai Lake. Relative dietary contribution was estimated using relative density (RD) derived from fecal microhistological analysis. Less abundant plant families were grouped as “Others”.

At the family level, the diets of both herbivores were dominated by Poaceae, Cyperaceae, and Asteraceae, although their relative contributions varied among subpopulations (Figure 2; Table S3). Poaceae was the dominant family in both herbivores. Cyperaceae generally contributed more to the diet of domestic sheep, whereas Asteraceae was relatively more represented in Przewalski’s gazelle. In addition, Fabaceae, Rosaceae, and Ephedraceae also tended to contribute more to the diet of Przewalski’s gazelle. Overall, both herbivores showed a predominantly graminoid-based dietary composition, while Przewalski’s gazelle incorporated a broader range of non-graminoid taxa across subpopulations. Detailed genus- and species-level relative density data are provided in Table S4.

### 3.2. Diet Diversity at Family, Genus, and Species Levels

We calculated Shannon diversity (*H*′) and Pielou’s evenness (*J*) for Przewalski’s gazelle and domestic sheep across the eight sympatric subpopulations (Table 2). Shannon diversity was significantly higher in Przewalski’s gazelle than in domestic sheep at all three taxonomic levels. At the family level, *H*′ ranged from 1.462 to 1.986 for gazelles and from 1.173 to 1.624 for sheep (paired-samples *t*-test, *t*_7_ = 8.18, *p* < 0.001). At the genus level, *H*′ ranged from 2.174 to 2.547 for gazelles and from 1.877 to 2.256 for sheep (*t*_7_ = 7.59, *p* < 0.001). At the species level, *H*′ ranged from 2.260 to 2.547 for gazelles and from 1.975 to 2.256 for sheep (*t*_7_ = 7.91, *p* < 0.001). For the pooled summaries based on mean relative density across the eight subpopulations, *H*′ values for Przewalski’s gazelle and domestic sheep were 1.854 and 1.420 at the family level, 2.759 and 2.477 at the genus level, and 2.911 and 2.580 at the species level, respectively.

Pielou’s evenness showed a similar overall pattern. At the family level, *J* ranged from 0.635 to 0.799 for gazelles and from 0.564 to 0.677 for sheep (*t*_7_ = 4.76, *p* = 0.002). At the genus level, *J* ranged from 0.791 to 0.850 for gazelles and from 0.751 to 0.814 for sheep (*t*_7_ = 4.36, *p* = 0.003). At the species level, *J* ranged from 0.821 to 0.857 for gazelles and from 0.751 to 0.822 for sheep (*t*_7_ = 4.50, *p* = 0.003). The corresponding pooled *J* values were 0.641 and 0.501 at the family level, 0.770 and 0.715 at the genus level, and 0.784 and 0.732 at the species level for Przewalski’s gazelle and domestic sheep, respectively.

**Figure 3:**
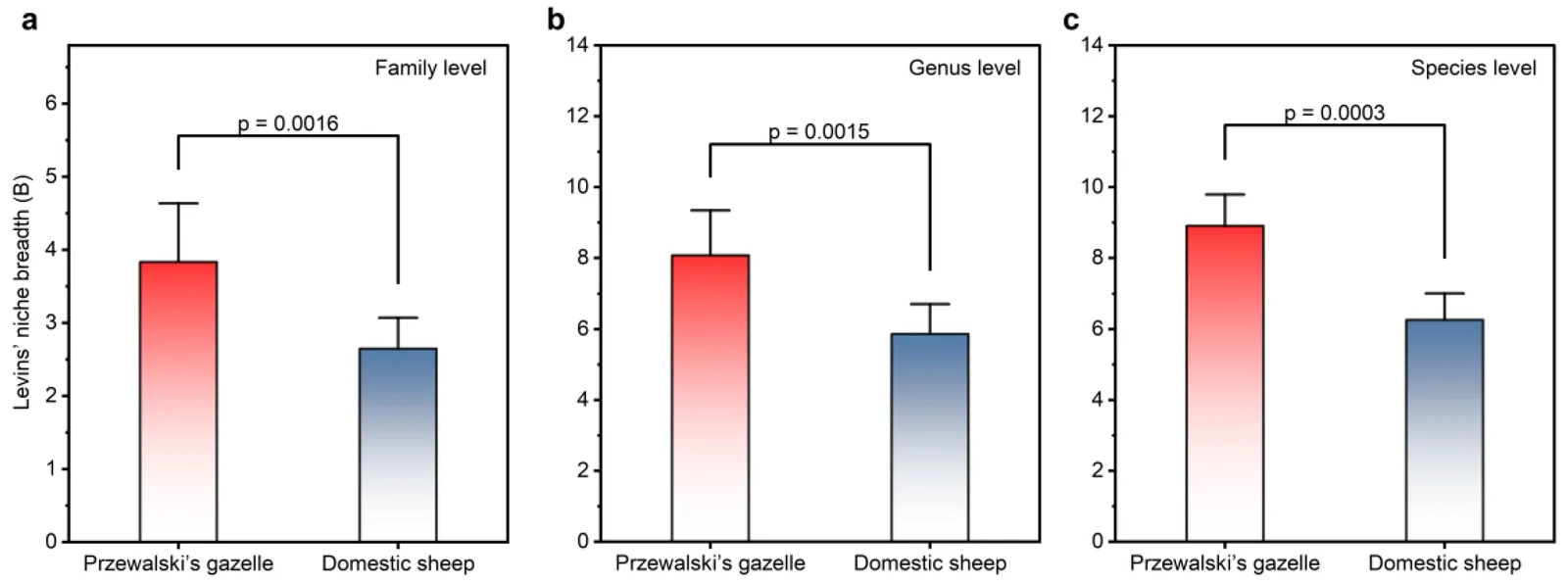
Levins’ niche breadth (*B*) of Przewalski’s gazelle and domestic sheep at the family (**a**), genus (**b**), and species (**c**) levels across eight sympatric subpopulations around Qinghai Lake. Red bars represent Przewalski’s gazelle, and blue bars represent domestic sheep. Bars represent mean ± SD across eight subpopulations. Differences between the two herbivores were tested using paired *t*-tests.

We also calculated Levins’ niche breadth (*B*) to assess the range of plant taxa exploited (Figure 3). At the family level, *B* ranged from 2.81 to 5.22 for gazelles and from 2.21 to 3.56 for sheep, with mean ± SD values of 3.83 ± 0.80 and 2.65 ± 0.42, respectively (paired-samples *t*-test, *t*_7_ = 4.96, *p* = 0.0016). At the genus level, *B* ranged from 6.80 to 10.22 for gazelles and from 4.60 to 7.40 for sheep, with mean ± SD values of 8.08 ± 1.27 and 5.86 ± 0.84, respectively (*t*_7_ = 5.06, *p* = 0.0015). At the species level, *B* ranged from 7.26 to 10.22 for gazelles and from 4.88 to 7.40 for sheep, with mean ± SD values of 8.91 ± 0.88 and 6.26 ± 0.75, respectively (*t*_7_ = 6.63, *p* = 0.0003).

### 3.3. Dietary Overlap Between Przewalski’s Gazelle and Domestic Sheep

We quantified dietary overlap between Przewalski’s gazelle and domestic sheep at the family, genus, and species levels using the numbers of shared and unique taxa and Pianka’s overlap index (Table 3). At the family level, the number of shared families ranged from 5 to 11 across subpopulations, with 17 shared families detected when records were pooled across all eight subpopulations. Przewalski’s gazelle had 1–4 unique families per subpopulation, whereas domestic sheep had 0–2 unique families. Pianka’s overlap index at the family level ranged from 0.830 to 0.976.

At the genus level, the number of shared genera ranged from 9 to 15 per subpopulation, with 32 shared genera detected in the pooled records. Przewalski’s gazelle had 2–10 unique genera per subpopulation, whereas domestic sheep had 0–3 unique genera. Pianka’s overlap index ranged from 0.718 to 0.946. At the species level, the number of shared species ranged from 10 to 15 per subpopulation, with 34 shared species detected in the pooled records. Przewalski’s gazelle had 3–10 unique species per subpopulation, whereas domestic sheep had 0–3 unique species. Pianka’s overlap index ranged from 0.629 to 0.946.

Several widely shared forage species included *Agropyron cristatum*, *Kobresia humilis*, *Poa pratensis*, and *Artemisia frigida*. Other species, such as *Ephedra monosperma* and *Hippophae tibetana*, showed relatively greater use by Przewalski’s gazelle (Table S5). Overall, dietary overlap was generally higher at the family level than at the genus and species levels, with substantial variation among subpopulations.

## 4. Discussion

Our results show that Przewalski’s gazelle and domestic sheep across sympatric subpopulations around Qinghai Lake shared a common dietary foundation but differed in the breadth and fine-scale structure of resource use. Both herbivores relied predominantly on similar major plant groups, whereas Przewalski’s gazelle generally showed broader taxonomic coverage, higher dietary diversity, and broader niche breadth than domestic sheep. Dietary overlap was generally higher at the family level and lower at the genus and species levels, with additional variation among subpopulations. Together, these findings characterize the dietary relationship between the two herbivores as a shared resource base accompanied by fine-scale differences in dietary resource use.

### 4.1. Shared Dietary Foundation with Broader Taxonomic Coverage in Gazelles

Przewalski’s gazelle and domestic sheep relied predominantly on the same major grassland plant groups, particularly Poaceae, Cyperaceae, and Asteraceae, resulting in generally high dietary overlap at the family level. Liu and Jiang (2004) identified graminoids, composites, legumes, sedges, and Rosaceae as major dietary components of both herbivores [21]. Li et al. (2008) reported that domestic sheep mainly consumed grasses and sedges, whereas legumes and composites were more prominent in the diets of Przewalski’s gazelle [16]. These previous findings support the importance of these major plant groups in the diets of both herbivores, although their relative contributions may vary among studies. Together, these results indicate that the two herbivores share a common dietary foundation within sympatric grasslands.

Despite this shared dietary foundation, fecal microhistological analysis identified a broader range of plant taxa in Przewalski’s gazelle samples than in domestic sheep samples, particularly at the genus and species levels. This pattern suggests that the apparent dietary relationship between the two herbivores depends partly on the taxonomic resolution used for comparison. Similar differences between dietary overlap at broad forage-category and finer plant-species levels have been reported in other sympatric ungulate systems [30,31,32]. The occurrence of this pattern across multiple sympatric subpopulations around Qinghai Lake suggests that the dietary relationship between Przewalski’s gazelle and domestic sheep cannot be adequately characterized by coarse taxonomic overlap alone. Importantly, broader taxonomic coverage does not necessarily indicate that the observed differences were caused by interspecific competition. The two herbivores may still share a common dietary base while differing in the representation of some finer-scale plant taxa in their fecal samples.

### 4.2. Greater Dietary Diversity and Broader Niche Breadth in Gazelles

Przewalski’s gazelle showed higher Shannon diversity, Pielou’s evenness, and Levins’ niche breadth than domestic sheep at the family, genus, and species levels. These results indicate that, at the subpopulation level, gazelle diets encompassed a broader and more even range of plant resources, whereas sheep diets were more concentrated on a smaller number of dominant forage resources.

The broader dietary breadth of Przewalski’s gazelle may arise from several non-exclusive mechanisms, including species-specific foraging strategies, differences in resource selectivity, and responses to local resource availability [33]. As managed livestock, domestic sheep may also be influenced by grazing management and husbandry, whereas gazelles are not subject to such direct husbandry control. However, these factors were not quantified in the present study, and the observed differences in dietary breadth should not be attributed directly to livestock management or interspecific competition.

Comparable patterns have been reported in other alpine and high-elevation grazing systems, where wild ungulates can exhibit broader dietary niches than sympatric livestock under some seasonal or ecological conditions [34,35]. A study of yaks on the Qinghai–Tibetan Plateau has likewise documented strong reliance on dominant graminoid resources [36]. In this context, the higher dietary diversity and niche breadth of Przewalski’s gazelle suggest greater flexibility in resource use. Such flexibility may be particularly relevant for a species persisting in fragmented subpopulations and exposed to spatially variable habitat conditions and livestock grazing [7,8].

### 4.3. Scale-Dependent Overlap and Spatial Heterogeneity

Another important finding of this study is that dietary overlap was generally higher at the family level and lower at the genus and species levels. This pattern suggests that Przewalski’s gazelle and domestic sheep appeared more similar when diets were compared at broad taxonomic resolution, whereas finer taxonomic resolution showed lower apparent overlap in resource use [29]. Our results show that the dietary relationship between the two herbivores is scale-dependent, with the extent of apparent overlap varying according to the taxonomic resolution considered [37]. Previous evaluations of fecal microhistological analysis have shown that the method can provide useful estimates of major dietary components, although taxon-specific identification difficulties and biases in estimated dietary proportions may occur [38,39]. In the present study, plant identification was supported by reference slides prepared from botanist-identified local plants, with representative diagnostic microstructures of reference materials and corresponding fecal fragments presented in Table S2. These procedures provided a morphological basis for taxonomic identification but did not constitute independent validation of the dietary estimates. Therefore, the lower dietary overlap estimated at the genus and species levels should be interpreted with caution, given the uncertainties associated with fine-scale microhistological identification.

Dietary overlap also varied among sympatric subpopulations, indicating substantial spatial heterogeneity in the dietary relationship between gazelles and sheep within the Qinghai Lake Basin. Some subpopulations showed relatively high overlap, whereas others showed markedly lower overlap, suggesting that the degree of dietary similarity is not spatially uniform across the region. Such variation may reflect differences in local plant-community composition, resource availability, grazing pressure, or habitat structure [9,40,41]. However, these factors were not directly quantified in the present study, so the underlying causes of among-subpopulation variation cannot be determined. Although both herbivores occurred within the same study areas, the degree of fine-scale habitat-use overlap was not quantified. Therefore, their spatial co-occurrence should not be interpreted as evidence of simultaneous use of the same forage patches. Nevertheless, the observed spatial heterogeneity suggests that the dietary relationship between Przewalski’s gazelle and domestic sheep varies among local ecological settings.

### 4.4. Conservation and Management Implications

Conservation planning for Przewalski’s gazelle and sympatric grasslands in the Qinghai Lake region should consider more than grassland area [42]. Forage composition and diversity also matter. Shared core species such as *Agropyron cristatum* and *Kobresia humilis* formed part of the common forage base. Gazelles also used a wider range of non-graminoid plants, suggesting that maintaining plant-community diversity may be important rather than focusing only on a few dominant forage species [43,44].

Dietary overlap varied substantially among subpopulations, so management should account for local conditions. Areas with relatively high dietary overlap may warrant closer monitoring of forage availability and livestock use, particularly where the two herbivores rely on similar dominant forage resources [44]. Maintaining a diverse forage base may also help preserve alternative food resources available to Przewalski’s gazelle. Importantly, dietary overlap alone does not demonstrate competition [45]; management decisions should therefore consider dietary information alongside forage availability, grazing pressure, and gazelle population status, particularly during the pre-green-up period.

### 4.5. Limitations

This study has several limitations. Fecal samples were pooled within each herbivore and subpopulation, so the dietary metrics represent subpopulation-level composite diets and cannot distinguish individual dietary variation or specialization. Plant taxa can differ in detectability and fragment preservation in fecal microhistological analysis, potentially introducing taxon-specific biases in reconstructed diet composition [38,39]. Although reference slides and standardized identification procedures helped maintain consistency, potential observer bias and uncertainty in genus- and species-level identification cannot be completely excluded. As independent molecular validation was not conducted, fine-scale dietary estimates should be interpreted with caution, and future studies combining fecal microhistology with DNA metabarcoding could help assess their reliability.

Forage availability, livestock grazing intensity, habitat-use overlap, the nutritional quality of the diets of both herbivores, gazelle body condition, and other local ecological factors were not quantified concurrently with dietary sampling. We therefore cannot link among-subpopulation variation in dietary overlap to specific environmental drivers, and dietary overlap alone should not be interpreted as evidence of interspecific competition [45]. Future studies combining individual-level dietary data with vegetation availability and quantitative grazing-pressure measurements would help identify the drivers of the observed patterns. Assessing the nutritional quality of forage consumed by both herbivores and monitoring seasonal changes in gazelle body condition (e.g., using standardized visual scoring), particularly during the pre-green-up period, would also help evaluate potential nutritional constraints.

## 5. Conclusions

We compared the diets of eight Przewalski’s gazelle subpopulations and sympatric domestic sheep around Qinghai Lake during the late winter grazing season (April 2023) at the family, genus, and species levels. The two herbivores shared a common dietary foundation dominated by major grassland plant groups but differed in the breadth and fine-scale structure of resource use. Fecal microhistological analysis revealed a broader range of identified plant taxa in Przewalski’s gazelle samples and generally higher estimates of dietary diversity, evenness, and niche breadth than in domestic sheep samples. Dietary overlap was generally higher at the family level and lower at the genus and species levels, indicating that the apparent similarity between the two herbivores depends on the taxonomic resolution considered. Dietary overlap also varied substantially among subpopulations, highlighting spatial heterogeneity in their dietary relationship across the region. These findings show that a shared dietary foundation can coexist with fine-scale differences in resource use and provide an ecological basis for conserving forage resource diversity and managing sympatric grasslands.

## Figures and Tables

**Table 1:** Taxonomic coverage of the diets of Przewalski’s gazelle (PG) and domestic sheep (DS) at the family, genus, and species levels across eight sympatric subpopulations.

Subpopulation	Family PG *n* (%)	Family DS *n* (%)	Family Total	Genus PG *n* (%)	Genus DS *n* (%)	Genus Total	Species PG *n* (%)	Species DS *n* (%)	Species Total
HN	14 (93.33)	11 (73.33)	15	21 (91.30)	17 (73.91)	23	21 (91.30)	17 (73.91)	23
HS	12 (100.00)	8 (66.67)	12	20 (100.00)	14 (70.00)	20	20 (100.00)	14 (70.00)	20
GN	10 (83.33)	8 (66.67)	12	19 (90.48)	11 (52.38)	21	20 (90.91)	12 (54.55)	22
GS	12 (100.00)	11 (91.67)	12	19 (95.00)	16 (80.00)	20	19 (95.00)	16 (80.00)	20
QF	11 (91.67)	8 (66.67)	12	17 (94.44)	14 (77.78)	18	18 (94.74)	15 (78.95)	19
TL	9 (81.82)	7 (63.64)	11	15 (83.33)	12 (66.67)	18	17 (85.00)	13 (65.00)	20
SI	9 (90.00)	9 (90.00)	10	14 (93.33)	13 (86.67)	15	16 (94.12)	14 (82.35)	17
HD	10 (90.91)	7 (63.64)	11	14 (87.50)	12 (75.00)	16	15 (88.24)	12 (70.59)	17
Pooled	18 (100.00)	17 (94.44)	18	36 (100.00)	32 (88.89)	36	41 (100.00)	34 (82.93)	41

PG = Przewalski’s gazelle; DS = domestic sheep. *n* indicates the number of plant taxa detected in the diet of each herbivore, and % represents the proportion of the total taxa recorded within each subpopulation that were detected in the diet of each herbivore. Total represents the total number of plant families, genera, or species detected in either herbivore within each subpopulation. Pooled represents the union of taxa detected across all eight subpopulations.

**Table 2:** Shannon diversity (*H*′) and Pielou’s evenness (*J*) of plant taxa consumed by Przewalski’s gazelle and domestic sheep across eight sympatric subpopulations.

Subpopulation	Family				Genus				Species			
	Shannon (*H*′)		Pielou’s (*J*)		Shannon (*H*′)		Pielou’s (*J*)		Shannon (*H*′)		Pielou’s (*J*)	
	PG	DS	PG	DS	PG	DS	PG	DS	PG	DS	PG	DS
HN	1.880	1.396	0.712	0.582	2.499	2.128	0.821	0.751	2.499	2.128	0.821	0.751
HS	1.765	1.299	0.71	0.624	2.547	2.083	0.85	0.789	2.547	2.083	0.850	0.789
GN	1.554	1.218	0.675	0.586	2.330	1.908	0.791	0.796	2.501	2.040	0.835	0.821
GS	1.986	1.624	0.799	0.677	2.456	2.256	0.834	0.814	2.456	2.256	0.834	0.814
QF	1.595	1.173	0.665	0.564	2.375	2.013	0.838	0.763	2.417	2.225	0.836	0.822
TL	1.679	1.173	0.764	0.603	2.204	1.877	0.814	0.755	2.402	1.975	0.848	0.77
SI	1.492	1.293	0.679	0.589	2.174	2.028	0.824	0.791	2.376	2.128	0.857	0.806
HD	1.462	1.292	0.635	0.664	2.211	2.013	0.838	0.810	2.260	2.013	0.835	0.810
Pooled	1.854	1.420	0.641	0.501	2.759	2.477	0.770	0.715	2.911	2.580	0.784	0.732

PG = Przewalski’s gazelle; DS = domestic sheep. *H*′ = Shannon diversity index; *J* = Pielou’s evenness index. The pooled row was calculated from the mean relative density of each taxon across the eight subpopulations, with each subpopulation weighted equally.

**Table 3:** Dietary overlap between Przewalski’s gazelle and domestic sheep at the family, genus, and species levels across eight sympatric subpopulations.

Subpopulation	Family				Genus				Species			
	Shared	PG-Only	DS-Only	Pianka	Shared	PG-Only	DS-Only	Pianka	Shared	PG-Only	DS-Only	Pianka
HN	10	4	1	0.954	15	6	2	0.887	15	6	2	0.887
HS	8	4	0	0.942	14	6	0	0.791	14	6	0	0.791
GN	6	4	2	0.830	9	10	2	0.768	10	10	2	0.739
GS	11	1	0	0.976	15	4	1	0.946	15	4	1	0.946
QF	7	4	1	0.963	13	4	1	0.849	14	4	1	0.840
TL	5	4	2	0.891	9	6	3	0.718	10	7	3	0.629
SI	8	1	1	0.962	12	2	1	0.821	13	3	1	0.827
HD	6	4	1	0.917	10	4	2	0.790	10	5	2	0.796
Pooled	17	1	0	–	32	4	0	–	34	7	0	–

“Shared” indicates the number of plant taxa consumed by both herbivores in each subpopulation; “PG-only” and “DS-only” indicate taxa consumed exclusively by Przewalski’s gazelle or domestic sheep, respectively. Pianka’s overlap index ranges from 0 (no overlap) to 1 (complete overlap). The “Pooled” row represents the union of taxa detected across all eight subpopulations; Pianka’s overlap index was not calculated for the pooled data.

## Data Availability

The original contributions presented in this study are included in the article/Supplementary Material. Further inquiries can be directed to the corresponding author.

## Supplementary Materials

The following supporting information can be downloaded at: https://www.mdpi.com/article/10.3390/ani16213426/s1. Figure S1: Representative photographs of fecal pellets from (a) Przewalski’s gazelle and (b) domestic sheep collected in the Qinghai Lake Basin; Table S1: Number of fecal deposits sampled from Przewalski’s gazelle and domestic sheep across eight study sites in the Qinghai Lake Basin; Table S2: Representative epidermal microstructures of plant species and corresponding fragments identified in fecal samples; Table S3: Family-level relative density (RD) of plant taxa in the diets of Przewalski’s gazelle and domestic sheep across eight sympatric subpopulations; Table S4: Genus- and species-level relative density (RD) of plant taxa in the diets of Przewalski’s gazelle and domestic sheep across eight sympatric subpopulations; Table S5: Species-level classification of dietary resources of Przewalski’s gazelle and domestic sheep across eight sympatric subpopulations.
