# Supplementary material for: Comparative Analysis of Diet Composition and Dietary Diversity in Wild Przewalski’s Gazelle and Sympatric Domestic Sheep Around Qinghai Lake, Western China
Source: Animals (Basel). 2026 Oct 29;16(21):3426. doi: 10.3390/ani16213426 (PMC13761968; doi:10.3390/ani16213426)
Supplement: Supplementary file 1 [file animals-16-03426-s001.zip › animals-4606028-supplementary.pdf]

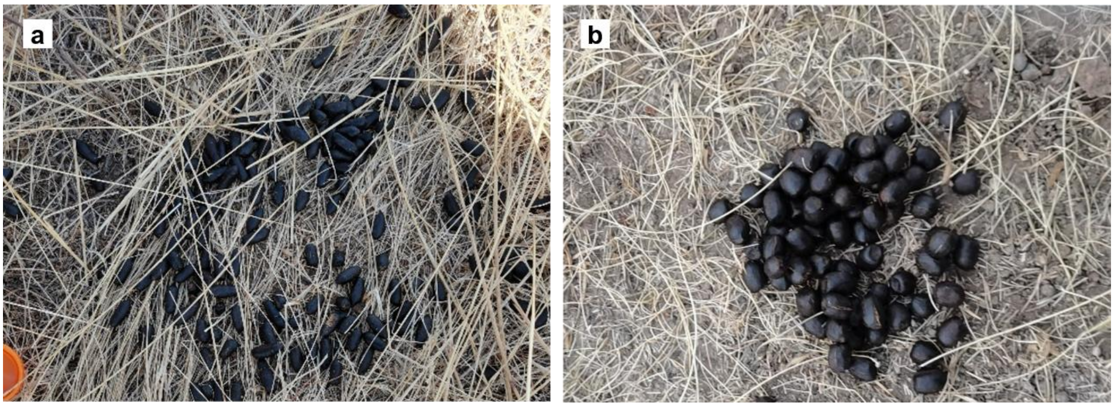

Figure S1. Representative photographs of fecal pellets from (a) Przewalski's gazelle and (b) domestic sheep collected in the Qinghai Lake Basin.

Table S1. Number of fecal deposits sampled from Przewalski's gazelle and domestic sheep across eight study sites in the Qinghai Lake Basin.

Subpopulation	Przewalski's gazelle	Domestic sheep
HN	8	5
HS	8	5
GN	8	8
GS	8	7
QF	8	8
TL	8	5
SI	5	5
HD	6	5
Total	59	48

Table S2 Representative epidermal microstructures of plant species and corresponding fragments identified in fecal samples.

Family	Plant species	Microscopic view (plants)	Microscopic view (feces)
Poaceae	*Poa pratensis*	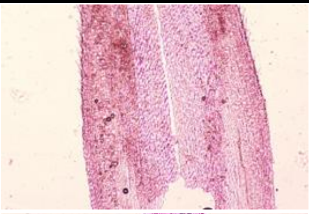	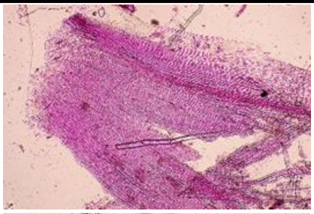
	Elymus nutans	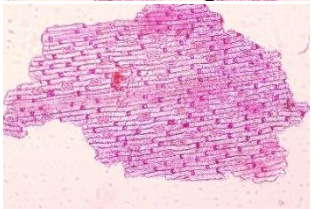	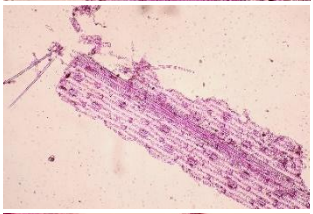
	Agropyron cristatum	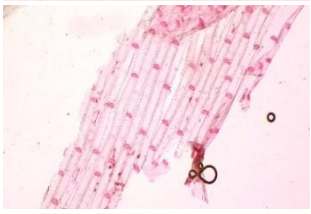	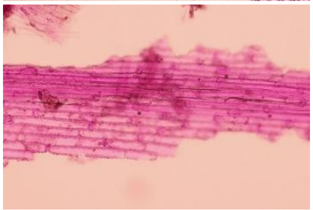
	Achnatherum splendens	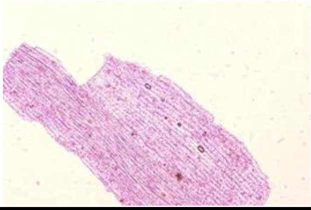	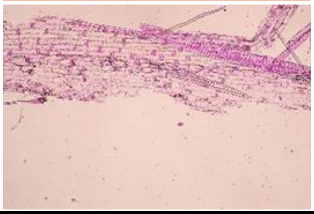
Cyperaceae	*Kobresia humilis*	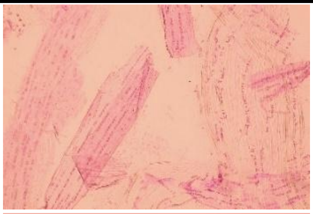	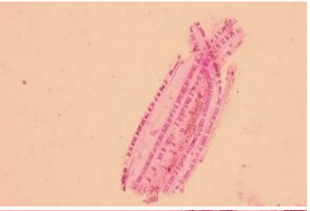
	Carex orbicularis	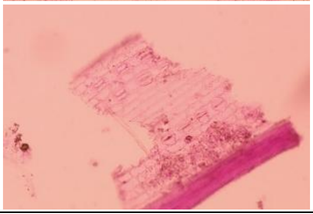	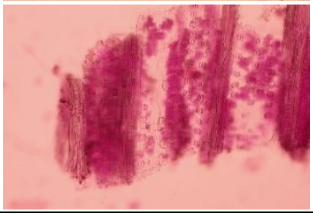
Asteraceae	*Aster altaicus*	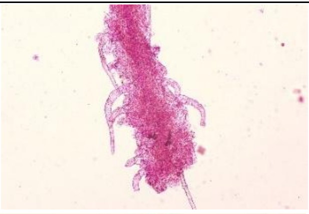	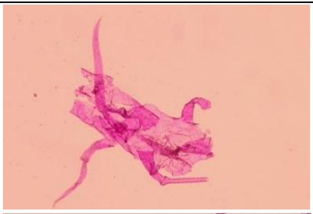
	Artemisia frigida	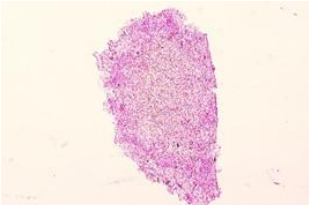	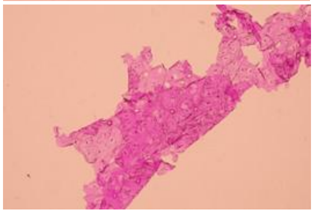

Family	Plant species	Microscopic view (plants)	Microscopic view (feces)
	Taraxacum mongolicum	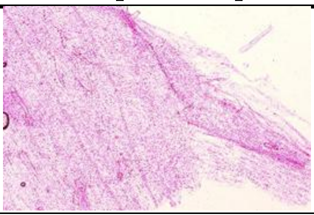	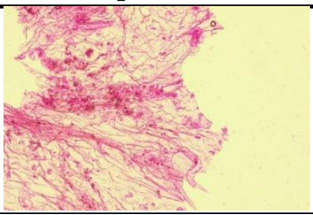
	Astragalus polycladus	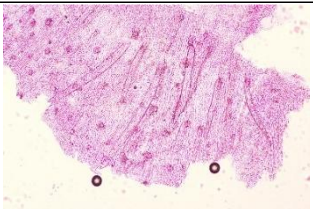	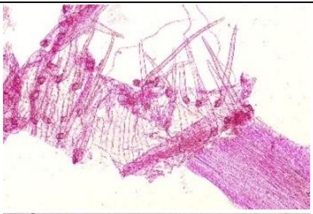
Fabaceae	*Oxytropis stracheyana*	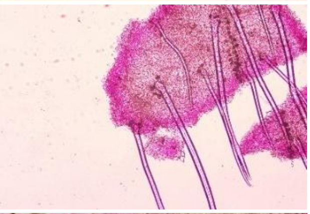	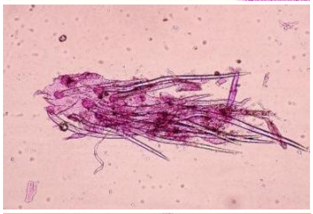
	Oxytropis falcata	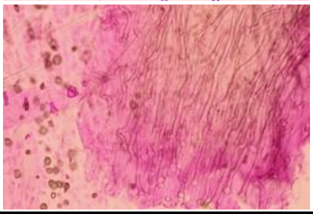	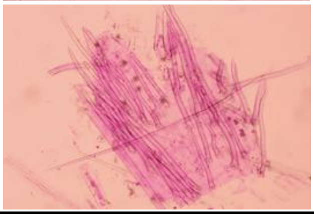
	Potentilla multifida	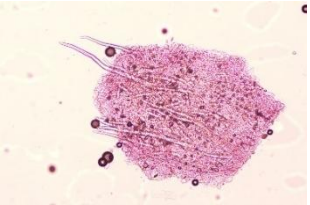	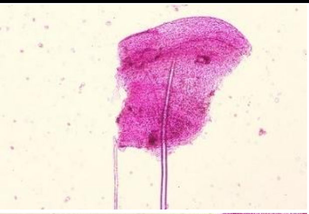
Rosaceae	*Sibbaldianthe bifurca*	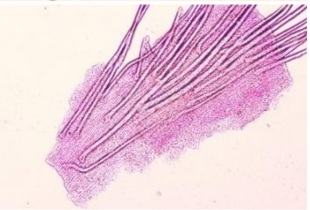	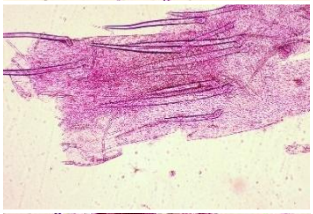
	Sibbaldianthe adpressa	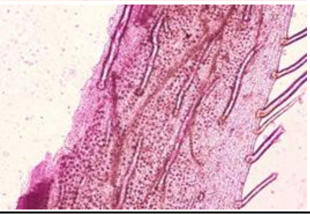	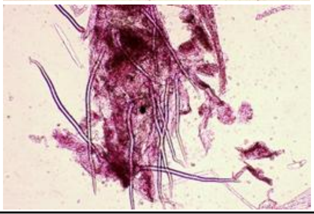
Others	*Hippophae tibetana*	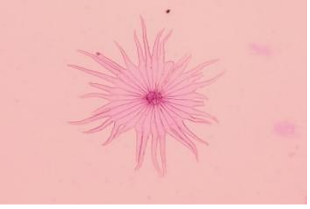	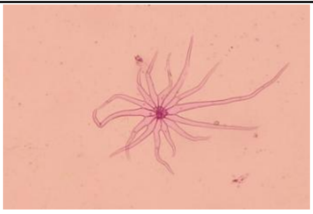

Family	Plant species	Microscopic view (plants)	Microscopic view (feces)
	Allium przewalskianum	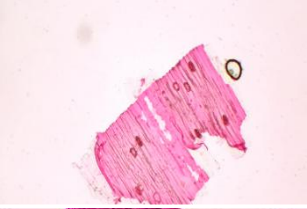	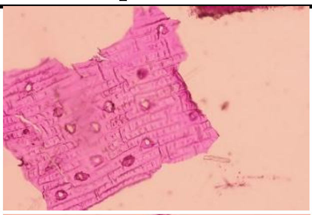
	Dracocephalum heterophyllum	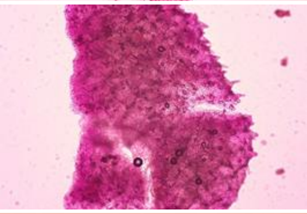	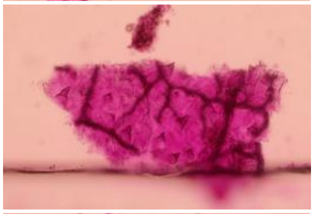
	Stellera chamaejasme	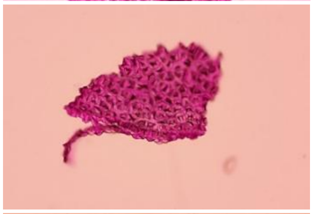	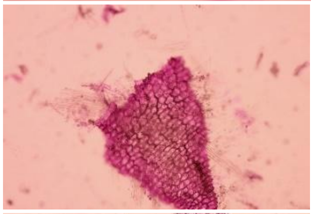
	Ephedra monosperma	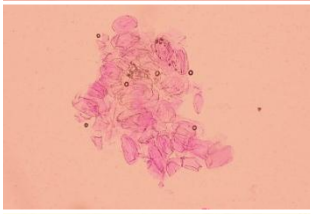	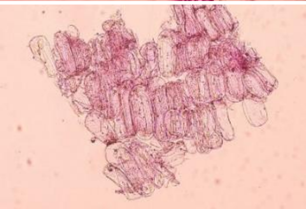
	Plantago depressa	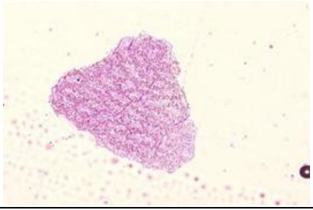	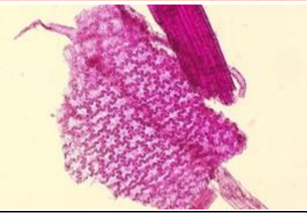

Table S3. Family-level relative density (RD) of plant taxa in the diets of Przewalski's gazelle and domestic sheep across eight sympatric subpopulations.

Plant family	RD (%)															
	HN		HS		GN		GS		QF		TL		SI		HD	
	PG	DS	PG	DS	PG	DS	PG	DS	PG	DS	PG	DS	PG	DS	PG	DS
Poaceae	42.33	56.44	43.02	56.95	45.94	47.42	35.23	46.2	52.43	63.91	35.81	64.81	46.74	58.99	55.96	53.62
Cyperaceae	10.19	20.43	8.33	20.73	9.54	37.49	18.99	19.97	8.66	15.68	20.48	13.3	13.99	18.39	6.47	26.62
Asteraceae	15.76	9.88	20.33	10.66	25.87	7.03	13.68	13.5	10.8	11.87	17.35	6.65	23.38	12.32	14.78	5.53
Fabaceae	3.85	2.1	6	0.6	7.88	3.72	5.28	4.11	2.26	2.61	11.16	9.57	7.28	1.79	1.23	–
Rosaceae	2.69	1.05	3.34	2.81	1.39	–	3.46	1.76	1.07	–	10.49	2.34	1.72	2.24	4.31	2.55
Scrophulariaceae	0.38	–	2.17	–	–	0.62	4.38	2.35	–	–	1.61	–	–	0.9	1.54	–
Apiaceae	3.27	2.94	–	–	–	–	2.56	1.96	3.73	1.66	–	–	–	–	–	–
Lamiaceae	0.58	–	0.5	–	2.35	–	1.82	0.59	–	–	0.4	–	–	–	0.77	–
Gentianaceae	1.54	0.63	–	–	–	–	–	–	2.4	–	–	–	–	–	–	–
Brassicaceae	2.88	0.42	0.67	–	2.07	–	0.55	–	1.07	0.95	–	0.78	1.34	1.13	1.23	–
Liliaceae	–	–	3.16	2.41	2.49	2.27	3.65	7.82	3.33	1.9	2.02	–	4.02	1.57	–	–
Elaeagnaceae	5.19	3.15	–	–	–	–	–	–	13.87	–	–	–	–	–	–	–
Plantaginaceae	0.58	–	2.5	1	–	–	–	–	–	–	–	–	–	–	–	–
Amaranthaceae	0.38	–	–	–	–	–	–	–	–	–	–	–	0.96	2.69	2.15	7.44
Primulaceae	–	–	–	–	2.07	–	–	–	–	–	–	–	–	–	–	–
Iridaceae	–	–	–	–	0.41	0.41	–	–	–	–	0.67	–	0.57	–	–	–
Thymelaeaceae	–	1.26	0.33	–	–	1.03	3.65	0.21	0.4	–	–	–	–	–	–	1.06
Ephedraceae	10.38	1.68	9.67	4.82	–	–	7.3	1.56	–	1.42	–	2.54	–	–	11.55	3.19

PG = Przewalski's gazelle; DS = domestic sheep; RD = relative density. Values represent the relative dietary contribution (%) of each plant family within each subpopulation. "–" indicates that the plant family was not detected in the diet.

Table S4. Genus- and species-level relative density (RD) of plant taxa in the diets of Przewalski's gazelle and domestic sheep across eight sympatric subpopulations.

Plant species	Plant genus	RD (%)															
		HN		HS		GN		GS		QF		TL		SI		HD	
		PG	DS	PG	DS	PG	DS	PG	DS	PG	DS	PG	DS	PG	DS	PG	DS
Poa pratensis	*Poa*	19.25	18.11	12.67	14.08	16.47	22.58	2.73	9.39	17.08	13.54	10.5	38.41	0	0	9.24	7.44
Elymus nutans	*Elymus*	–	–	1.83	3.01	–	–	–	–	–	–	–	–	–	–	–	–
Leymus secalinus	*Leymus*	8.84	12	10.67	28.6	–	–	10.77	15.27	3.33	6.88	–	–	–	–	–	–
Agropyron desertorum	*Agropyron*	–	–	–	–	11.07	8.28	–	–	1.07	9.5	17.1	3.91	16.48	9.64	–	–
Agropyron cristatum	*Agropyron*	14.24	24.86	17.85	11.26	13.84	11.18	21.73	21.54	20.02	27.82	8.21	15.65	11.11	5.6	24.99	17.66
**—**	***Agropyron***	**14.24**	**24.86**	**17.85**	**11.26**	**24.91**	**19.46**	**21.73**	**21.54**	**21.09**	**37.32**	**25.31**	**19.56**	**27.59**	**15.24**	**24.99**	**17.66**
Achnatherum splendens	*Achnatherum*	–	–	–	–	1.24	–	–	–	5.33	2.85	–	6.84	6.89	20.19	2.46	0.85
Orinus kokonorica	*Orinus*	–	–	–	–	–	–	–	–	–	–	–	–	12.26	23.56	19.27	22.99
Stipa purpurea	*Stipa*	–	1.47	–	–	3.32	5.38	–	–	5.6	3.32	–	–	–	–	–	4.68
Kobresia humilis	*Kobresia*	10.19	20.43	8.33	20.73	4.98	25.9	18.99	19.97	8.66	15.68	20.48	13.3	13.99	18.39	6.47	26.62
Carex arcatica	*Carex*	–	–	–	–	4.56	11.59	–	–	–	–	–	–	–	–	–	–
Artemisia frigida	*Artemisia*	11.15	6.73	7.5	5.43	16.33	7.03	9.67	8.22	9.2	8.31	12.38	4.89	12.65	7.17	6.93	1.7
Artemisia salsoloides	*Artemisia*	–	–	–	–	–	–	–	–	–	–	–	–	–	–	2.15	–
**—**	***Artemisia***	**11.15**	**6.73**	**7.5**	**5.43**	**16.33**	**7.03**	**9.67**	**8.22**	**9.2**	**8.31**	**12.38**	**4.89**	**12.65**	**7.17**	**9.08**	**1.7**
Aster altaicus	*Aster*	2.5	2.52	10.5	4.02	9.13	–	1.64	2.74	1.6	3.56	2.15	–	10.73	5.15	5.7	3.83
Taraxacum mongolicum	*Taraxacum*	0.96	0.42	2.33	1.21	0.41	–	2.37	2.54	–	–	–	–	–	–	–	–
Leontopodium nanum	*Leontopodium*	1.15	0.21	–	–	–	–	–	–	–	–	2.82	1.76	–	–	–	–
Astragalus polycladus	*Astragalus*	3.27	2.1	4.33	0.6	5.53	3.72	4.19	3.13	0.53	1.66	3.09	6.84	2.49	0.67	1.23	–
Oxytropis stracheyana	*Oxytropis*	–	–	1.67	–	1.66	–	–	0.98	–	–	4.84	–	0.77	–	–	–
Oxytropis falcata	*Oxytropis*	–	–	–	–	–	–	–	–	–	–	1.75	1.56	1.91	–	–	–
**—**	***Oxytropis***	**–**	**–**	**1.67**	**–**	**1.66**	**–**	**–**	**0.98**	**–**	**–**	**6.59**	**1.56**	**2.68**	**–**	**–**	**–**

Thermopsis lanceolata	*Thermopsis*	0.58	–	–	–	0.69	–	1.09	–	1.73	0.95	1.48	1.17	2.11	1.12	–	–
Potentilla multifida	*Potentilla*	0.77	1.05	1.67	0.8	1.11	–	2.55	1.76	1.07	–	3.5	2.34	–	–	–	–
Sibbaldianthe bifurca	*Sibbaldianthe*	1.92	–	0.5	2.01	–	–	0.91	–	–	–	6.99	–	–	–	4.31	2.55
Sibbaldianthe adpressa	*Sibbaldianthe*	–	–	–	–	0.28	–	–	–	–	–	–	–	–	–	–	–
**—**	***Sibbaldianthe***	**1.92**	**–**	**0.5**	**2.01**	**0.28**	**–**	**0.91**	**–**	**–**	**–**	**6.99**	**–**	**–**	**–**	**4.31**	**2.55**
Argentina anserina	*Argentina*	–	–	1.17	–	–	–	–	–	–	–	–	–	1.72	2.24	–	–
Pedicularis kansuensis	*Pedicularis*	0.38	–	–		–	–	–	–	–	–	–	–	–	–	–	–
Pedicularis alaschanica	*Pedicularis*	–	–	–	–	–	0.62	–	–	–	–	1.61	–	–	0.9	1.54	–
**—**	***Pedicularis***	**0.38**	**–**	**–**	**–**	**–**	**0.62**	**–**	**–**	**–**	**–**	**1.61**	**–**	**–**	**0.9**	**1.54**	**–**
Lagotis brachystachya	*Lagotis*	–	–	2.17	–	–	–	4.38	2.35	–	–	–	–	–	–	–	–
Bupleurum pusillum	*Bupleurum*	3.27	2.94	–	–	–	–	2.01	1.96	3.73	1.66	–	–	–	–	–	–
Carum carvi	*Carum*	–	–	–	–	–	–	0.55	–	–	–	–	–	–	–	–	–
Dracocephalum heterophyllum	*Dracocephalum*	0.58	–	0.5	–	2.35	–	1.82	0.59	–	–	0.4	–	–	–	0.77	–
Gentiana dahurica	*Gentiana*	1.54	0.65	–	–	–	–	–	–	2.4	–	–	–	–	–	–	–
Lepidium apetalum	*Lepidium*	–	–	0.67	–	–	–	–	–	–	–	–	–	–	–	–	–
Braya humilis	*Braya*	2.88	0.42	–	–	2.07	0	0.55	–	1.07	0.95	–	0.78	1.34	1.13	1.23	–
Allium przewalskianum	*Allium*	–	–	3.16	2.41	2.49	2.27	3.65	7.82	3.33	1.9	2.02	–	4.02	1.57	–	–
Hippophae tibetana	*Hippophae*	5.19	3.15	–	–	–	–	–	–	13.87	–	–	–	–	–	–	–
Plantago depressa	*Plantago*	0.58	–	2.5	1	–	–	–	–	–	–	–	–	–	–	–	–
Chenopodium album	*Chenopodium*	0.38	–	–	–	–	–	–	–	–	–	–	–	–	–	–	–
Salsola collina	*Salsola*	–	–	–	–	–	–	–	–	–	–	–	–	0.96	2.69	2.15	7.44
Androsace mariae	*Androsace*	–	–	–	–	2.07	–	–	–	–	–	–	–	–	–	–	–
Iris lactea	*Iris*	–	–	–	–	0.41	0.41	–	–	–	–	0.67	–	0.57	–	–	–
Stellera chamaejasme	*Stellera*	–	1.26	0.33	–	–	1.03	3.65	0.21	0.4	–	–	–	–	–	–	1.06
Ephedra monosperma	*Ephedra*	10.38	1.68	9.67	4.82	–	–	7.3	1.56	–	1.42	–	2.54	–	–	11.55	3.19

Note: PG, Przewalski’s gazelle; DS, domestic sheep. A dash (–) in the RD data columns indicates that the corresponding plant taxon was not detected in the diet samples. A dash (—) in the “Plant species” column indicates a genus-level RD value, calculated by summing the RD values of all identified species within the same genus. For genera represented by a single identified species, genus-level RD values are identical to species-level RD values.

Table S5 Species-level classification of dietary resources of Przewalski's gazelle and domestic sheep across eight sympatric subpopulations.

Family	Plant species	Przewalski's gazelle		Domestic sheep		Categories
		n	Mean RD (%)	n	Mean RD (%)	
Poaceae	*Agropyron cristatum*	8	16.48	8	16.95	Shared core
Cyperaceae	*Kobresia humilis*	8	11.50	8	20.13	Shared core
Poaceae	*Poa pratensis*	7	10.99	7	15.44	Shared core
Asteraceae	*Artemisia frigida*	8	10.72	8	6.19	Shared core
Asteraceae	*Aster altaicus*	8	5.49	6	2.73	Shared core
Fabaceae	*Astragalus polycladus*	8	3.08	7	2.34	Shared core
Liliaceae	*Allium przewalskianum*	6	2.33	5	2	Shared core
Poaceae	*Elymus nutans*	1	0.23	1	0.38	Shared secondary
Poaceae	*Leymus secalinus*	4	4.19	4	7.84	Shared secondary
Poaceae	*Agropyron desertorum*	4	5.72	4	3.92	Shared secondary
Poaceae	*Achnatherum splendens*	4	1.99	4	3.84	Shared secondary
Poaceae	*Orinus kokonorica*	2	3.94	2	5.82	Shared secondary
Poaceae	*Stipa purpurea*	2	1.12	4	1.86	Shared secondary
Cyperaceae	*Carex arcatica*	1	0.57	1	1.45	Shared secondary
Asteraceae	*Taraxacum mongolicum*	4	0.76	3	0.52	Shared secondary
Asteraceae	*Leontopodium nanum*	2	0.5	2	0.25	Shared secondary
Fabaceae	*Oxytropis stracheyana*	4	1.12	1	0.12	Shared secondary
Fabaceae	*Thermopsis lanceolata*	6	0.96	3	0.41	Shared secondary
Fabaceae	*Oxytropis falcata*	2	0.46	1	0.2	Shared secondary
Rosaceae	*Potentilla multifida*	6	1.33	4	0.74	Shared secondary
Rosaceae	*Sibbaldianthe bifurca*	5	1.83	2	0.57	Shared secondary
Rosaceae	*Argentina anserina*	2	0.36	1	0.28	Shared secondary
Scrophulariaceae	*Pedicularis alaschanica*	2	0.39	2	0.19	Shared secondary
Scrophulariaceae	*Lagotis brachystachya*	2	0.82	1	0.29	Shared secondary

Apiaceae	*Bupleurum pusillum*	3	1.13	3	0.82	Shared secondary
Lamiaceae	*Dracocephalum heterophyllum*	6	0.8	1	0.07	Shared secondary
Gentianaceae	*Gentiana dahurica*	2	0.49	1	0.08	Shared secondary
Brassicaceae	*Braya humilis*	6	1.14	4	0.41	Shared secondary
Elaeagnaceae	*Hippophae tibetana*	2	2.38	1	0.39	Shared secondary
Plantaginaceae	*Plantago depressa*	2	0.39	1	0.13	Shared secondary
Amaranthaceae	*Salsola collina*	2	0.39	2	1.27	Shared secondary
Iridaceae	*Iris lactea*	3	0.21	1	0.05	Shared secondary
Thymelaeaceae	*Stellera chamaejasme*	3	0.54	4	0.45	Shared secondary
Ephedraceae	*Ephedra monosperma*	4	4.86	6	1.9	Shared secondary
Asteraceae	*Artemisia salsoloides*	1	0.27	0	0	Gazelle-exclusive
Rosaceae	*Sibbaldianthe adpressa*	1	0.04	0	0	Gazelle-exclusive
Scrophulariaceae	*Pedicularis kansuensis*	1	0.05	0	0	Gazelle-exclusive
Apiaceae	*Carum carvi*	1	0.07	0	0	Gazelle-exclusive
Brassicaceae	*Lepidium apetalum*	1	0.08	0	0	Gazelle-exclusive
Amaranthaceae	*Chenopodium album*	1	0.05	0	0	Gazelle-exclusive
Primulaceae	*Androsace mariae*	1	0.26	0	0	Gazelle-exclusive

PG = Przewalski’s gazelle; DS = domestic sheep. n indicates the number of sympatric subpopulations in which each plant species was detected in the diet of each herbivore. Mean RD represents the mean relative density of each species across all eight subpopulations, with non-detections treated as zero. Species recorded in at least five of the eight subpopulations in both herbivores and with a mean RD ≥ 2% in at least one herbivore were classified as shared core resources. Species detected in both herbivores but not meeting these criteria were classified as shared secondary resources. Species detected only in Przewalski’s gazelle or domestic sheep were classified as gazelle-exclusive or sheep-exclusive, respectively.
